# Recent Advances in Tetra-Coordinate Boron-Based Photoactive Molecules for Luminescent Sensing, Imaging, and Anticounterfeiting

**DOI:** 10.1021/prechem.4c00072

**Published:** 2024-12-06

**Authors:** Dingfang Hu, Rongrong Huang, Yu Fang

**Affiliations:** Key Laboratory of Applied Surface and Colloid Chemistry of Ministry of Education, School of Chemistry and Chemical Engineering, Shaanxi Normal University, Xi’an 710119, P. R. China

**Keywords:** photoactive tetra-coordinate boron molecules, bidentate chelating ligand, coordination atom, photophysical properties, electron delocalization, luminescent sensing, anticounterfeiting, luminescent imaging

## Abstract

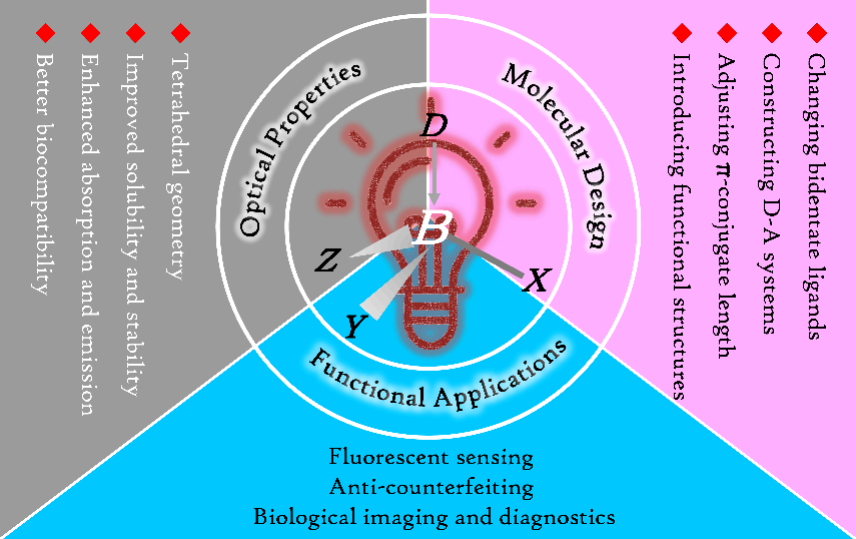

Tetra-coordinate boron-based fluorescent materials hold considerable promise across chemistry, biology and materials science due to their unique and precisely tunable optoelectronic properties. The incorporation of the heteroatom boron (B) enables these materials to exhibit high luminescence quantum yields, adjustable absorption and emission wavelengths, and exceptional photostability. This review examines the molecular design and applications of tetra-coordinate boron-based photoactive molecules, highlighting their roles in fluorescence sensing, anticounterfeiting, and imaging. We outline how structural features impact their properties and discuss strategies for enhancing their performance, including ligand modification and the extension of conjugation length, among others. Additionally, future research focus in this field is also addressed including strategies for diversifying molecular structures and enhancing molecular stability, which is believed to pave the way for innovative solutions to the challenges in areas such as sensing, imaging and information security.

## 1 Introduction

**Scheme 1:**
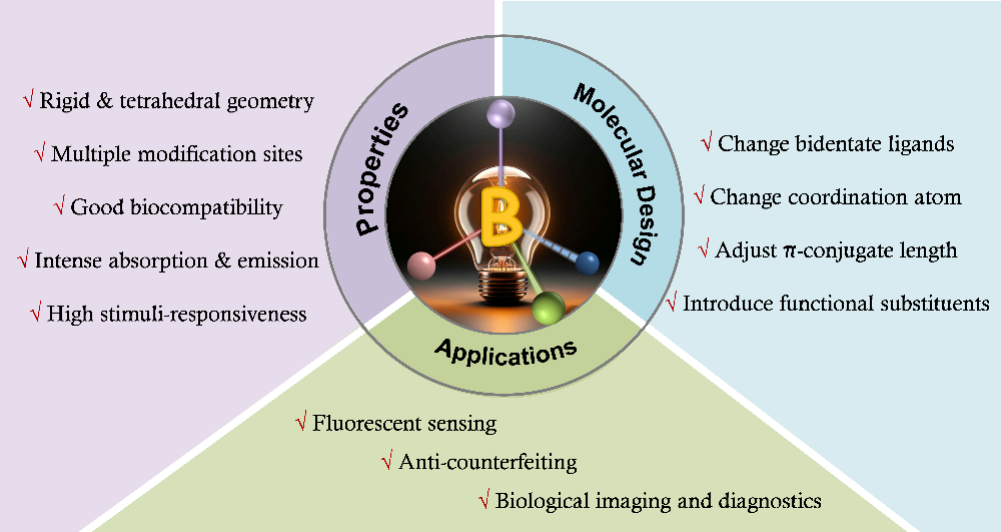
Overview of Topics on Tetra-Coordinate Boron-Based Photoactive Materials

Luminescent materials are attracting growing interest in the fields of chemistry, biology, and materials science due to their diverse and tunable photophysical properties.^1−4^ Recent advancements in scientific and technological developments have positioned the design and application of functional fluorescent materials as a key area of research. Among these materials, tetra-coordinate boron-based molecules have emerged as a captivating class of compounds in organic chemistry, drawing significant attention for their unique electronic properties and versatile applications.^5−7^ The incorporation of boron atom (B) into organic scaffolds introduces distinct structural and electronic characteristics, leading to pronounced photophysical properties, such as high quantum yields, tunable emission wavelengths and excellent photostability. These characteristics make tetra-coordinate boron compounds highly attractive for various technological applications, including luminescence sensing, anticounterfeiting and luminescence imaging (Scheme 1).

The distinctive properties of tetra-coordinate boron-based photoactive molecules arise primarily from the electron-deficient nature of the boron center, which facilitates efficient interactions with conjugated systems, and thereby enhances electron delocalization and charge transfer processes. These interactions are critical for the design of functional fluorescent materials, as they could enable precise modulation of optical properties through structural modifications.^8−13^ Modifications of the boron center with various conjugation systems allow for the effective modulation of molecular orbital energy levels, enabling the customization and tuning of luminescent properties to meet specific requirements. Yamaguchi and co-workers modulated the electronic structure and lowered the lowest unoccupied molecular orbital (LUMO) levels of fully fused ladder-type π-conjugated frameworks via the utilization of tetra-coordinated boron as a bridging unit.^14^ In the molecular design, the intramolecular B–N coordination effectively fixes the π-conjugated framework in a planar fashion and substantially affects the electronic structure through an increase in electron affinity.

The luminescent properties of tetra-coordinate boron-based photoactive molecules can also be enriched by integrating with various excited-state photophysical processes (e.g., intramolecular charge transfer, energy transfer, proton transfer, intersystem crossing), endowing these compounds with various environmental sensitivity.^15^ In addition, the rigid tetrahedral geometry of the tetra-coordinate boron moiety provides an excellent building block that facilitates high luminescent efficiency in solid state, due to the screening of unfavorable electronic interactions from dense intermolecular interactions. The tetrahedral stereochemical structure also facilitates a high mass transfer process which is believed to improve the sensing performances of solid materials.^16^ These features enable tetra-coordinate boron luminophores to be promising candidates for developing diverse functional materials for high-performance fluorescent sensing and anticounterfeiting.

Fluorescence imaging, particularly in biological systems, is another area where tetra-coordinate boron-based molecules have shown great promise.^17,18^ Through rational molecular modifications and integration with various photophysical processes, the luminescence properties (i.e., absorption and emission intensity, wavelength, and lifetime) of tetra-coordinate boron derivatives can be finely adjusted to fulfill particular application needs. For example, photoinduced electron transfer (PET) and intramolecular charge transfer (ICT) processes can be systematically tuned in boron-dipyrromethene (BODIPY) dyes by tethering different electron-withdrawing or donating groups at the 8′ and 5′ positions of the BODIPY core. Both the types and positions of the substituents show impacts on the push–pull strengths in the molecular system, enabling efficient modulation of the excited-state transition. This approach allows for precise modulation of their emission wavelength and lifetime, enabling high-performance imaging to distinguish multiple targets using fluorescence lifetime as a signal.^19^ Additionally, biocompatibility is another significant asset of tetra-coordinate boron-based materials. These materials generally exhibit minimal phototoxicity, good photostability and low cytotoxicity, making them suitable for *in vivo* imaging and other biological applications.

In this minireview, we will explore recent advances in the design and application of tetra-coordinate boron-based photoactive molecules. We will discuss the structural characteristics that influence their photophysical properties, emphasizing their emerging roles in luminescent sensing, anticounterfeiting, and luminescent imaging. Additionally, we will identify the challenges and future directions in this rapidly evolving field. Through this comprehensive overview, we aim to underscore the transformative potential of tetra-coordinated boron-based materials and inspire further research and innovation in this exciting area of chemistry.

## 2 Molecular Design and Properties

### 2.1 Structural Properties of Tetra-Coordinate Boron

**Figure 1:**
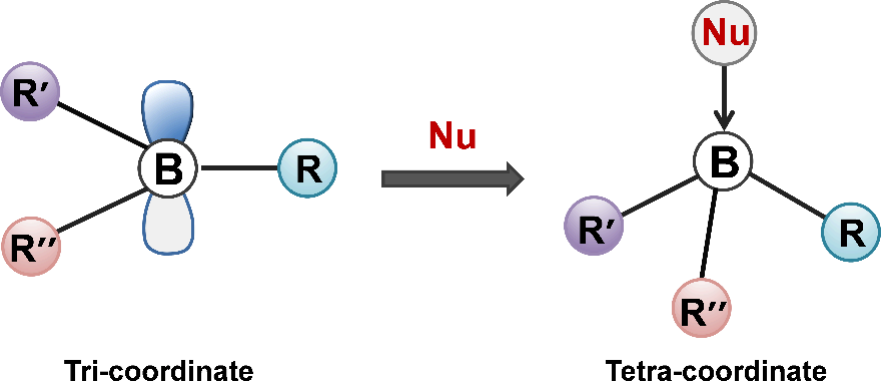
Schematic illustration for the tricoordinate organoboron compounds and the tetra-coordinate organoboron compounds. Nu represents nucleophile, the three substituents (R, R’, R’’) can be identical or differ from one another.

Boron, a unique nonmetallic element in group IIIA of the periodic table, possesses three valence electrons and two vacant *p*-orbitals. This electronic configuration allows boron to adopt two primary hybridization states: (i.e., sp^2^ and sp^3^). Tricoordinate boron compounds, formed via sp^2^ hybridization, usually exhibit significant Lewis acidity due to the presence of an unoccupied *p*_*z*_ orbital. This makes them prone to nucleophilic attack by atoms with lone electron pairs (e.g., nitrogen and oxygen atoms). When these nucleophiles donate electron pairs to boron, it leads to the formation of sp^3^-hybridized boron centers that adopt a stable tetrahedral geometry (Figure 1). This sp^3^ hybridization occupies the vacant *p*-orbitals, thereby decreasing the reactivity of the compounds and enhancing their stability against air and water. Consequently, tetra-coordinate boron compounds exhibit greater robustness compared to their tricoordinate counterparts.^20−24^

Additionally, the formation of intramolecular B-X (X: N or O) coordination bonds facilitates extensive electron delocalization throughout the molecule, resulting in a rigid π-conjugated framework. This rigidity suppresses energy loss through vibrations within the π-system, thereby significantly enhancing emission efficiency.^25−27^ Moreover, the molecular design lowers the LUMO level, which in turn increases the molecule’s electron affinity.

These structural characteristics endow tetra-coordinate boron-based organic luminescent materials with exceptional optoelectronic properties. They exhibit efficient light absorption and emission, capable of deep-red and near-infrared fluorescence. Additionally, these materials have excellent electron transport properties and can respond to various external stimuli, rendering them highly suitable for applications in fluorescence sensing, anticounterfeiting, and biological imaging. Owing to these advantageous properties, tetra-coordinate boron compounds have been extensively studied and applied in the development of fluorescent materials.

### 2.2 Strategies for Tunable Luminescence of Tetra-Coordinate Boron

The unique coordination structure of tetra-coordinate boron makes it an attractive candidate for building new materials with tunable properties. This section explores several key strategies to adjust electronic structures and precisely tailor the luminescent properties of tetra-coordinate boron compounds for targeted applications.

**Figure 2:**
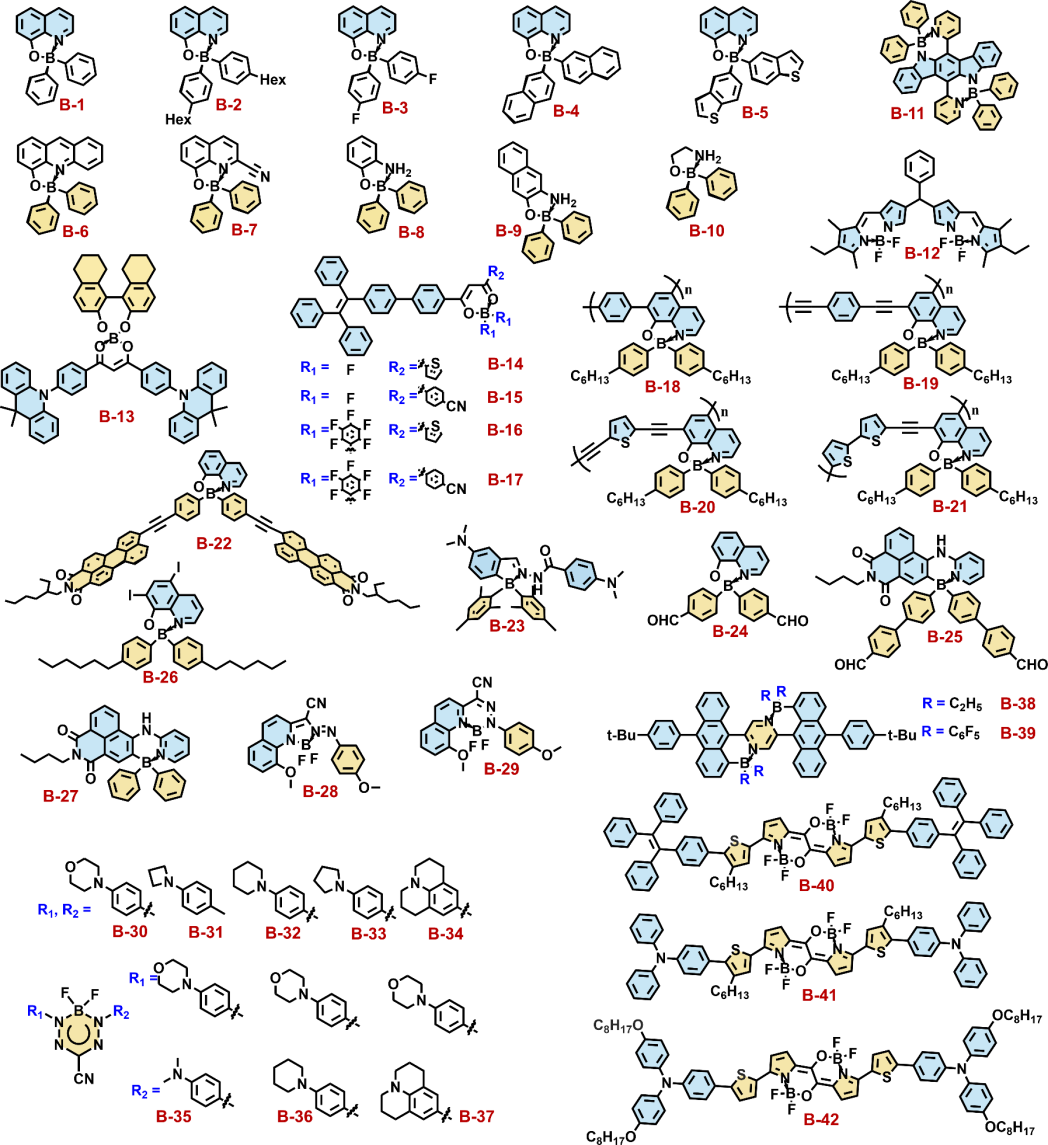
Chemical structures of the selected tetra-coordinate boron derivatives involved in this review.

Specifically, these strategies range from subtle adjustments to the ligand structure to innovative designs of the overall molecular configuration. The main approaches include: 1) modifying the bidentate chelating ligands to directly influence the electronic environment around the luminescent center; 2) adjusting the length of the conjugated backbone to control the energy range of light absorption and emission; 3) incorporating various functional substituents into the ligand to further enrich the properties and functionalities of the compounds; and 4) changing the types of atoms bonded to B to explore how synergistic effects originated from different elements impact the luminescence properties. These design strategies have led to the development of an extensive library of photoactive tetra-coordinate boron functional molecules (Figure 2).

**Figure 3:**
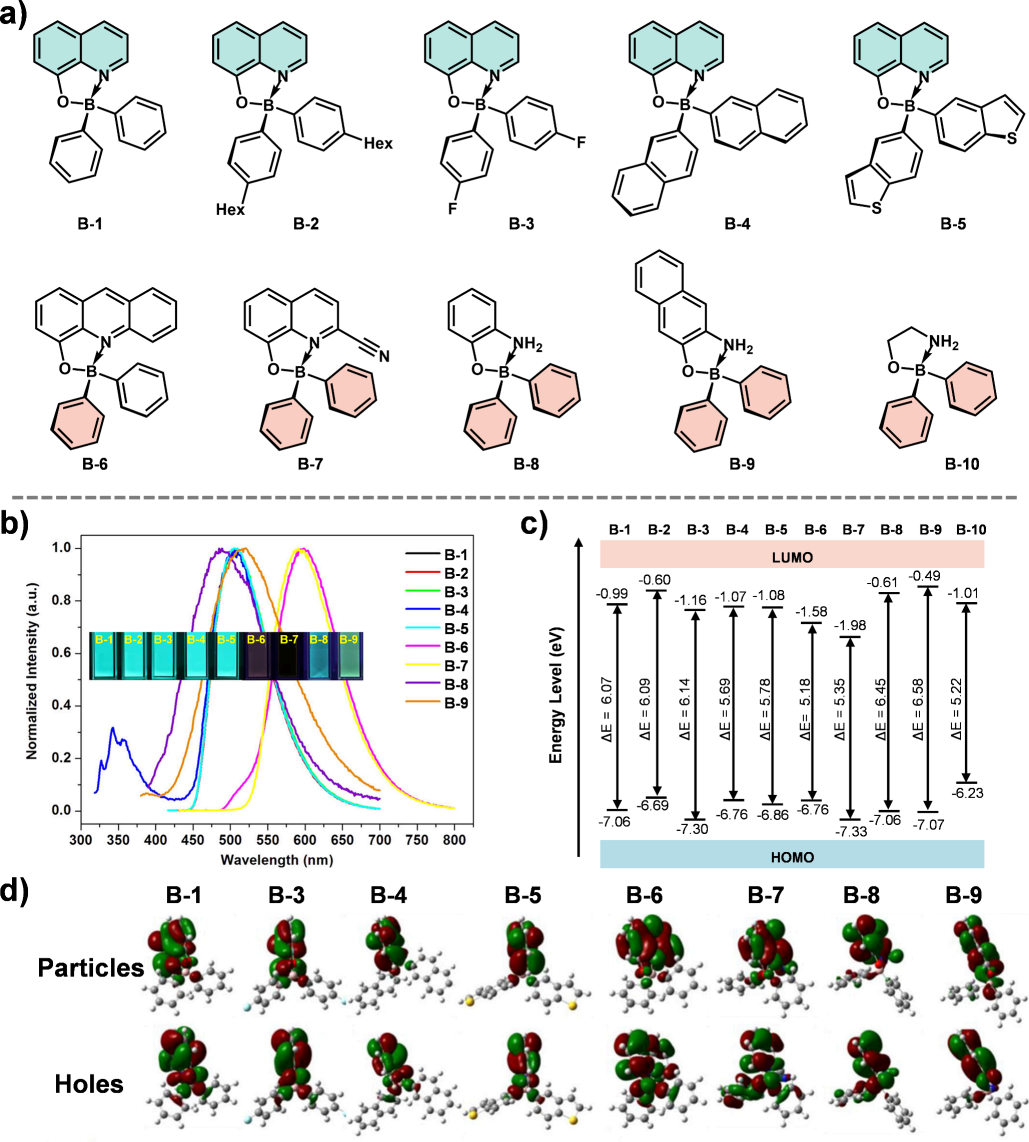
(a) Molecular structures of the as-prepared tetra-coordinate monoboron complexes. (b) Normalized fluorescence emission spectra of B-1 ∼ B-9 in THF (1.0 × 10^–6^ mol L^–1^). The inset is the optical image of the corresponding solutions under 365 nm UV light. (c) The highest occupied molecular orbital (HOMO) and lowest unoccupied molecular orbital (LUMO) levels of all the compounds at ground state balance geometry. (d) Dominant natural transition orbital pairs for the lowest singlet excited state geometry. Reproduced with permission.^28^ Copyright 2017 American Chemical Society.

Altering the electronic nature of bidentate chelating ligands is a powerful strategy for controlling the electron density distribution around the boron center, which directly impacts the photophysical properties. Qi et al. conducted a comprehensive study on the photophysical properties of a series of tetra-coordinate boron compounds based on 8-hydroxyquinoline ligand (Figure 3a).^28^ Their findings revealed that altering the monodentate ligands while keeping the bidentate ligands constant led to similar absorption wavelengths and molar extinction coefficients, with negligible differences in the emission spectra. However, changing the bidentate ligands resulted in significant spectral differences (Figure 3b). Theoretical calculations indicated that the primary electronic transitions in the excited state are localized on the bidentate ligands and the boron atom, with minimal influence from the monodentate ligands (Figures 3c and 3d). These results highlight the pivotal role of bidentate ligands in dictating the photophysical properties of organoboron compounds, a conclusion consistent with prior research from Wang,^29,30^ White,^31^ Jäkle,^32^ and others. This study underscores the importance of chelating ligand design in developing boron-based luminescent materials with tailored optical properties.

**Figure 4:**
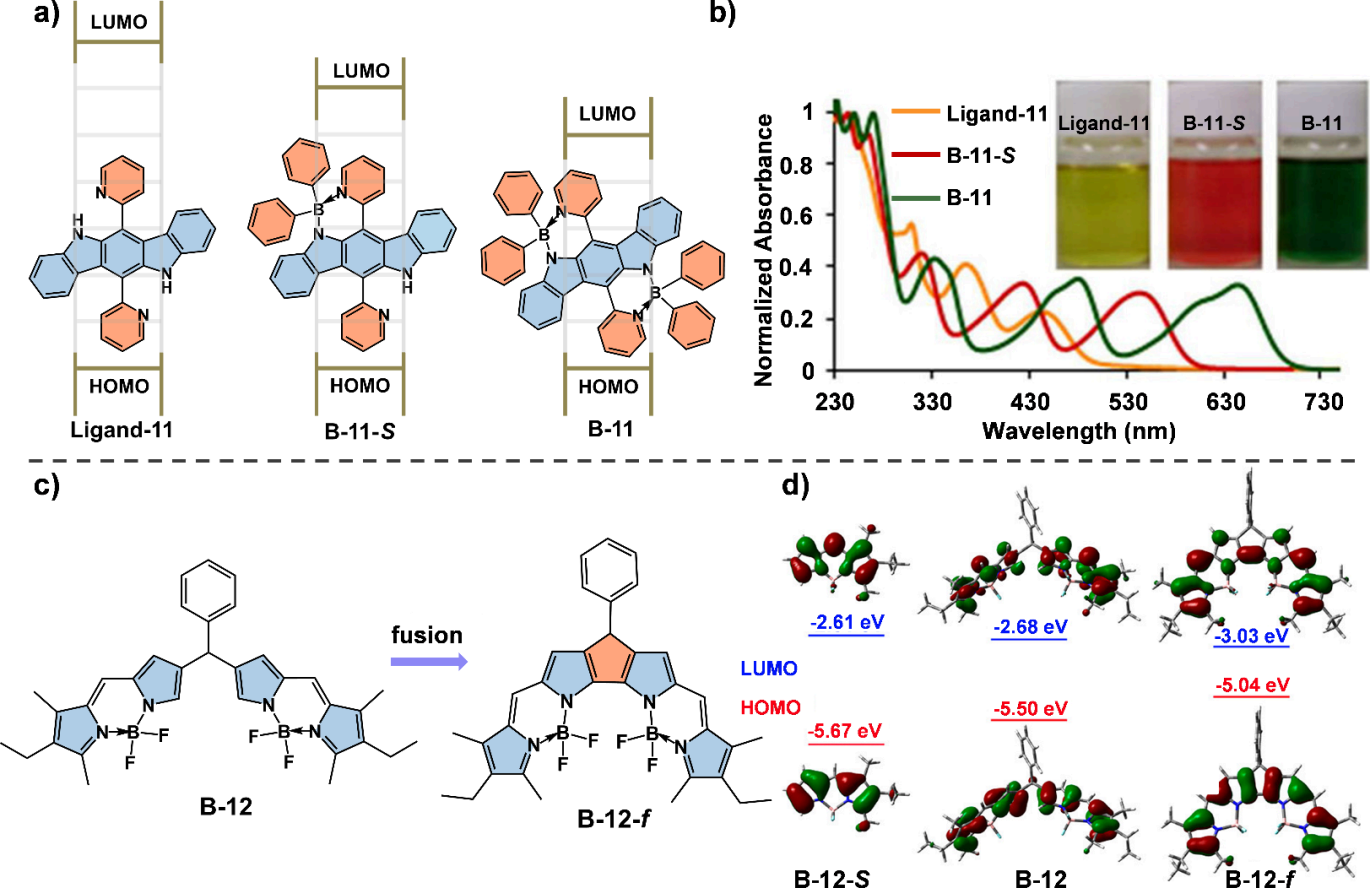
(a) Chemical structures of ligand and boron complexes and the corresponding energy levels. (b) Absorption spectra of compounds Ligand-11, B-11-*S*, and B-11 in CH_2_Cl_2_ (2 × 10^–5^ mol L^–1^). The insets are images of the three compounds under sunlight. Reproduced with permission.^33^ Copyright 2012 American Chemical Society. (c) Chemical structures of dimers and coplanar fused molecules of α, α-bisBODIPY. (d) Frontier molecular orbitals and their energy levels for B-12-*S*, B-12, and B-12-*f* from DFT calculations at the B3LYP/6-31G(d) level. Reproduced with permission.^34^ Copyright 2020 American Chemical Society.

The length of the conjugation system also affects the degree of electron delocalization, which in turn modulates the HOMO–LUMO gap. Extending the conjugation typically results in a redshift in luminescence, enabling emissions at longer wavelengths. This redshift is advantageous in applications requiring deeper tissue penetration, such as near-infrared (NIR) bioimaging. Furthermore, increased conjugation enhances the charge-carrier mobility within the molecule, which can significantly improve the performance of boron-based materials in photoelectric applications. Curiel et al. demonstrated the incorporation of multiple boron atoms into ladder-type conjugated systems,^33^ such as those based on indolo[3,2-*b*]carbazole, not only leads to a significant redshift in absorption but also enhances the stability (Figures 4a and 4b). The presence of boron centers helps to stabilize the LUMO level, thereby narrowing the HOMO–LUMO energy gap and improving the charge-transport capabilities. These modifications expand the potential of tetra-coordinate boron compounds for use in robust organic optoelectronic materials requiring high stability and efficiency in charge transport, such as field-effect transistors or photovoltaic cells.

Introducing structural constraints, such as five-membered rings to lock the conformation of BODIPY dimers, as shown in Jiao et al.’s study, effectively increases π-orbital overlap and electronic delocalization.^34^ This structural modification resulted in bis-BODIPYs (B-12-*f*) with a planar conformation and extended π-conjugation (Figure 4c). The conformational restriction effectively increased the overlap of π-orbitals between the BODIPY units, thereby enhancing the overall electronic delocalization (Figure 4d). As a result, these molecules exhibited strong absorption and emission in the NIR region, with absorption maxima around 760 nm and emission peaks around 780 nm, accompanied by high fluorescence quantum yields of up to 0.84. The strong NIR luminescence, coupled with excellent photostability, make the compounds particularly suitable for *in vitro* and *in vivo* fluorescence imaging applications, where deep tissue penetration, minimal photodamage and high signal-to-noise ratios are critical.

**Figure 5:**
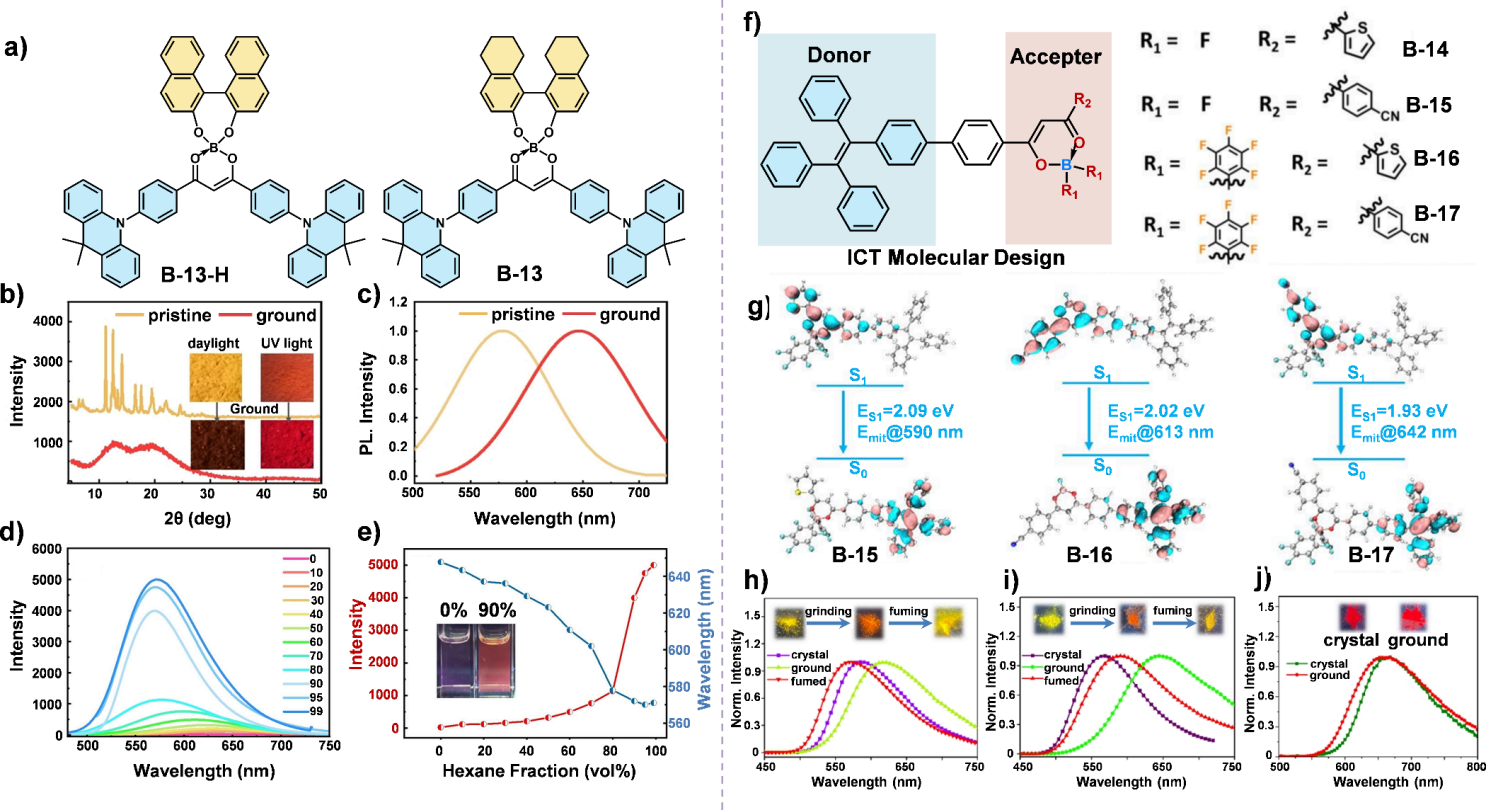
(a) Chemical structure of B-13-H and B-13. (b) Powder X-ray diffraction (PXRD) patterns of B-13-H before and after grinding. (c) Normalized emission spectra of B-13-H before and after grinding. (d) Emission spectra and (e) intensity change of B-13-H diluted in DCM/*n*-hexane mixtures with different *n*-hexane fractions. Reproduced with permission.^35^ Copyright 2022 Wiley-VCH. (f) Chemical structures of B-14 ∼ B-17. (g) The HOMO and LUMO distributions of B-15 ∼ B-17. Photoluminescence spectra of (h) B-15, (i) B-16 and (j) B-17 in various aggregated states under solid conditions. Reproduced with permission.^36^ Copyright 2024 Wiley-VCH.

In addition to the methods mentioned above, introducing functional substituents or modifying the bridging atoms in tetra-coordinate boron compounds can also significantly alter their photophysical properties. For example, Yang et al. successfully integrated chiral binaphthol or octa-hydro-binaphthol donors with tetra-coordinate boron acceptors, resulting in the formation of new enantiomers that exhibit unique optical behaviors (Figure 5a).^35^ These enantiomers displayed thermally activated delayed fluorescence (TADF) and circularly polarized luminescence (CPL), as well as piezochromism and aggregation-induced emission (AIE) characteristics (Figure 5b-5e). Such multifunctionality is attributed to the twisted configurations and chiral environment provided by the binaphthol moieties, which facilitate efficient spin–orbit coupling and exciton formation. By introducing bulky substituents with different electron-withdrawing abilities, Yan et al. developed a series of β-diketone boron complexes (B-14 ∼ B-17, Figure 5f).^36^ The ICT properties of these fluorophores were effectively modulated, resulting in emissions across a broad spectrum of wavelengths (Figure 5h-j). This strategy also enabled precise manipulation of solid-state packing, transforming the molecular arrangement from X-aggregates (B-15) to loosely packed structures (B-16) and tightly packed J-aggregates (B-17), endowing these complexes with outstanding piezochromic properties. These stimuli-responsive materials are promising in information encryption.

**Figure 6:**
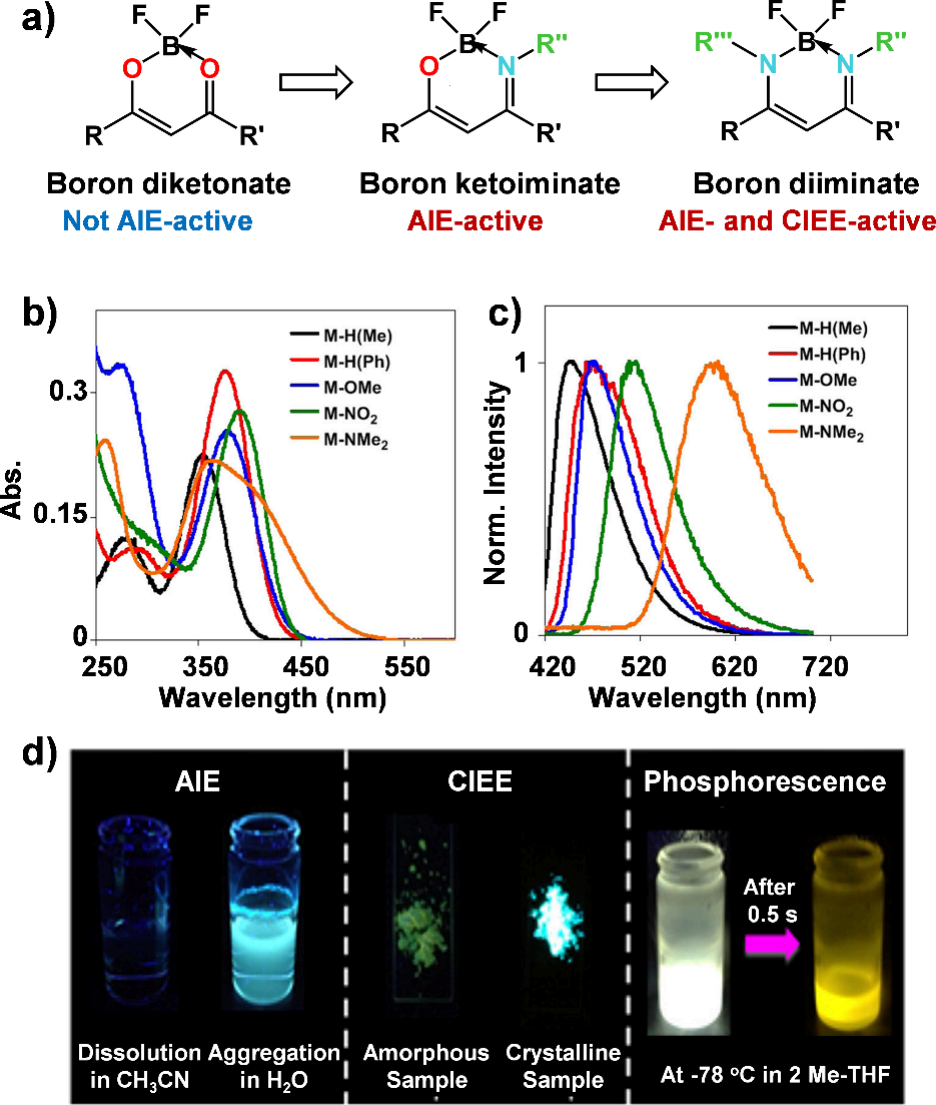
(a) Chemical structures of three boron derivatives. (b) UV–vis absorption spectra of the boron diiminate derivatives in THF (1.0 × 10^–5^ mol L^–1^). (c) Emission spectra of the synthesized boron diiminate derivatives in crystalline states (M represents the boron diiminate monomer compound). (d) Fluorescent images of the boron diiminate compounds exhibiting aggregation-induced emission, crystallization-induced emission, and phosphorescence properties. Reproduced with permission.^37^ Copyright 2014 American Chemical Society.

Furthermore, Chujo and his colleagues demonstrated that replacing oxygen atoms in β-diketonate ligands with nitrogen atoms led to the formation of boron-containing dyes with notable aggregation-induced emission (AIE) activity, a result of restricted intramolecular rotations that suppress nonradiative decay (Figures 6a-6c).^37,38^ Compared to the amorphous powder, the crystal exhibited significantly enhanced emission (Figure 6d), demonstrating a pronounced crystallization-induced emission enhancement (CIEE) effect. The improvement in emission was attributed to variations in intermolecular arrangements between the amorphous and crystalline states. This finding suggests that altering atoms bonded to the boron center can impart unique solid-state luminescent properties to tetra-coordinate boron compounds.

Overall, these strategies illustrate the intricate relationship between molecular structures and photophysical behaviors in tetra-coordinate boron compounds. For instance, bidentate ligand design and backbone conjugation not only tune absorption/emission wavelength but also influence charge transport, essential for optoelectronic applications. Functional substituents and atom replacement enable control over aggregation-induced behaviors, allowing these compounds to be tailored for stimuli-responsive solid-state luminescent materials.

This mechanistic understanding of the structure–property relationship can drive the development of boron-based materials with desired luminescent properties, opening pathways for their use in sensing, anticounterfeiting and bioimaging. Future research may focus on synergizing these strategies, such as combining extended conjugation with specific functional substituents, to create multifunctional materials that offer tunable emission and advanced luminescent behaviors. By advancing these approaches, luminescent materials with customized functionalities can be developed for targeted applications.

## 3 Applications

The functionalization strategies for tetra-coordinate boron compounds explored earlier not only deepen our understanding of their underlying luminescence mechanisms but also pave the way for the design of advanced molecular systems with tailored functionalities. By manipulating ligand structures, introducing specific functional groups, and altering bridging atoms, researchers can fine-tune the optical and electronic properties of these compounds. This ability to customize emission characteristics is crucial for developing materials suitable for a wide range of applications.

In the following section, we will delve into recent advancements in the applications of tetra-coordinate boron compounds, particularly in the areas of fluorescence sensing, anticounterfeiting, and bioimaging and diagnostics. These compounds are being leveraged for their unique optoelectronic properties such as high fluorescence quantum yields, tunable emission wavelengths, and strong photostability to develop sensitive and selective fluorescence sensors, secure anticounterfeiting materials, and enhance imaging techniques for medical diagnostics. By exploring these advancements, we aim to highlight the significant potential of tetra-coordinate boron compounds to drive innovation and solve critical challenges in scientific research and industry.

### 3.1 Fluorescence Sensing Applications

As a vital branch of optical sensing technology, fluorescence sensing has experienced significant advancements in both scientific research and industrial contexts in recent years.^39−41^ This technique relies on the ability of a molecule to emit light upon excitation at a specific wavelength. The emitted fluorescence can be quantitatively or qualitatively altered by interactions with target analytes, thereby providing a versatile method for detecting and quantifying various substances. The basic principle of fluorescence sensing involves the binding of a target analyte to a sensor molecule, which induces changes in the electronic structure and, consequently, the fluorescence properties of the sensor. These changes may manifest as shifts in emission wavelength, variations in intensity, or alterations in fluorescence lifetime. Owing to its high sensitivity, specificity, and rapid response, fluorescence sensing is widely employed in fields such as environmental monitoring, food safety, and chemical analysis.^42−46^ The effectiveness and mechanism of fluorescence sensors are directly influenced by the optical properties and stability of fluorescent molecules as well as their interactions with analytes. Therefore, a comprehensive evaluation of these factors is essential when selecting or designing fluorescent molecules.

Among various organic small molecules, tetra-coordinate boron compounds stand out due to their exhibit stimuli-responsive properties, which allow them to react to a wide range of environmental triggers such as light, heat, mechanical stress, and chemical vapors. These characteristics make them promising candidates for fluorescence sensing applications.

**Figure 7:**
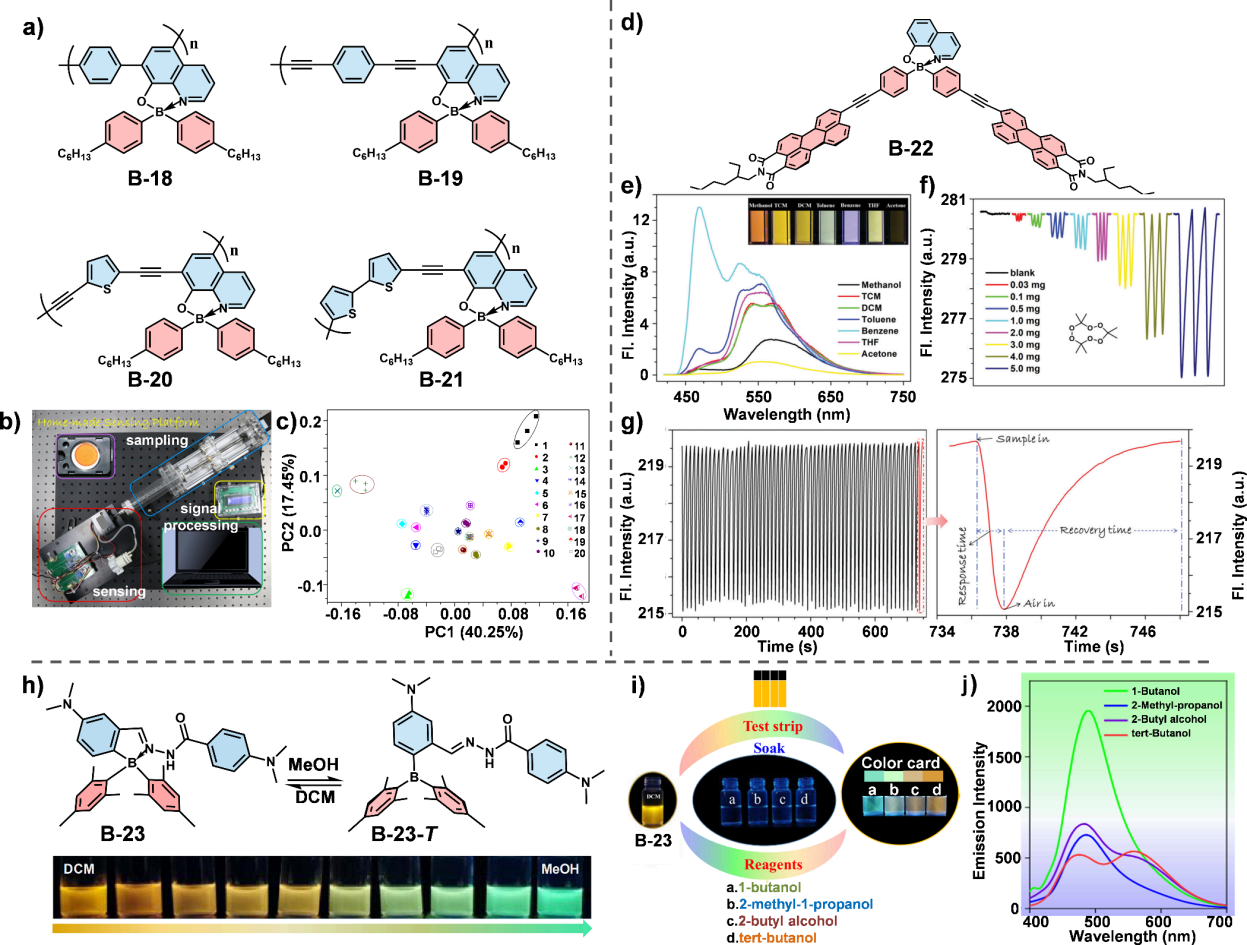
(a) Chemical structure of tetra-coordinate boron-based polymers. (b) Schematic diagram of self-made sensing platform. (c) Two-dimensional principal component analysis (PCA) score plot to discriminate the saturated vapors of the tested volatile small organic molecules (VSOMs) at 293 K. The Arabic numerals in the figure stand for different chemicals: methane (1), ethane (2), propane (3), *n*-butane (4), *n*-pentane (5), *n*-hexane (6), *n*-heptane (7), *n*-octane (8), *n*-nonane (9), *n*-decane (10), methanol (11), toluene (12), diethyl ether (13), benzene (14), acetone (15), ethanol (16), THF (17), DCM (18), TCM (19) and water (20). Reproduced with permission.^16^ Copyright 2018 The Royal Society of Chemistry. (d) Chemical structure of B-22. (e) Fluorescence emission spectra of B-22 in different solvents (C = 5.0 × 10^–6^ mol L^–1^) upon excitation at 400 nm. Inset is the relevant images taken under 365 nm UV light. (f) Response traces of the fluorescent B-22 film-based device to the presence of different amounts of TATP. (g) Reusability tests (60 times) of the fluorescent film-based device to 2600 ppm acetone vapor at ∼293 K (left), and the detailed meaning of the response traces (right). Reproduced with permission.^47^ Copyright 2019 The Royal Society of Chemistry. (h) Illustrations for the solvent-dependent molecular switches between B-23 and B-23-*T*. (i) Visual identification of butanol isomers with the test strip and reagents. (j) Fluorescence spectra of B-23 with the addition of butanol isomer (B-23 in DCM with a concentration of 1.0 × 10^–3^ mol L^–1^). Reproduced with permission.^48^ Copyright 2023 American Chemical Society.

Recently, our group reported the development of a conceptual fluorescence sensor array based on tetra-coordinate organoboron polymers (Figure 7a).^16^ The stereostructure of these molecules facilitates the formation of microchannels within the fluorescence sensing film at the molecular level. These microchannels allow analytes of different sizes, cohesive energies, and polarities to accumulate to varying extents, thereby enabling effective differentiation and identification. To evaluate the sensing performances of the tetra-coordinate organoboron polymers-based materials, a homemade platform capable of in situ, continuous online detection was developed, which consists of sampling, sensing, and signal processing components (Figure 7b). Four boron-containing conjugated polymers were selected to prepare sensitive films by dip-coating their solutions onto various substrates (i.e., silica plates and glass plates). In this way, a sensor array consisting of eight distinct films was fabricated, facilitating rapid and accurate identification of 20 common volatile organic compounds (VOCs) at room temperature (Figure 7c). This work provides valuable insights and strategies for the noninvasive diagnostics of critical diseases.

Furthermore, modifying the tetrahedral structure of 8-hydroxyquinolinato boron complex (BQ) with perylene monoamide (PMI) yielded the fluorescence sensing unit B-22, which shows high sensitivity to acetone and peroxide explosives such as triacetone triperoxide (TATP) (Figures 7d-7g).^47^ The quenching effect of these analytes on the sensor is attributed to the combined effects of “capillary condensation” and “solvation effect”. The nonplanar structure of B-22 prevents tight molecular stacking in the aggregated state, resulting in the formation of porous structures that increase the surface area. This increased surface area facilitates gas adsorption, a phenomenon known as the “capillary condensation” effect. Additionally, B-22 displays lower fluorescence quantum yield in acetone compared to other solvents and its solid-state form, indicating that the “solvation effect” plays a significant role in its selectivity for acetone and peroxide explosives.

These sensing behaviors primarily rely on changes in fluorescence intensity, which can be affected by factors such as variability in the light source and environmental interference, potentially compromising sensing accuracy. In contrast, ratiometric fluorescence sensing addresses these limitations by utilizing intensity changes across multiple emission bands. This method provides an internal calibration mechanism that effectively mitigates external fluctuations and interferences, significantly enhancing the precision and reliability of detection. Moreover, by carefully designing the emission color range, analytes can be visually distinguished based on fluorescence color changes, facilitating straightforward identification and quantification of target substances. Consequently, ratiometric fluorescence sensing not only improves the robustness and accuracy of the sensing process but also offers a versatile method for detecting a wide range of analytes in diverse and challenging environments.

Based on the principles of ratiometric sensing, Cao et al. developed a strategy using metastable Lewis acid–base pairs (B and N), to create multicolor fluorescent molecular switches (Figure 7h).^48^ By integrating Schiff base (C=N) groups into aminoborane frameworks and manipulating the B–N bonds through the use of alcohols, they were able to alter the mode of intramolecular charge transfer, resulting in a significant fluorescence shift from orange-red to green. The steric hindrances of the alkyl chains in the alcohols influenced the ratio of tricoordinated to tetra-coordinated boron compounds in B-23, allowing for the clear visual distinction of butanol isomers (Figure 7i). This capability to visually identify isomers demonstrates the potential of such molecular switches for simple, efficient and portable identification of various chemical species.

The performance of fluorescence sensing not only depends on the intrinsic properties of sensing units but also on the methods used to prepare the films. Traditional film-forming techniques, such as drop-casting and spin coating, often result in films with poor uniformity and weak substrate adhesion, which may negatively impact sensing performance due to uneven thickness and insufficient interaction with analytes.

**Figure 8:**
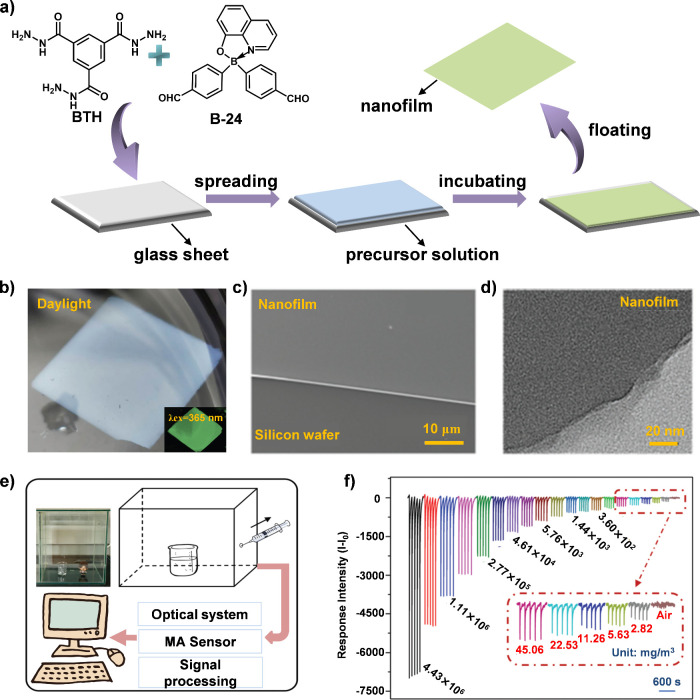
(a) Schematical illustrations for the structures of two building blocks (BTH and B-24), and the formation process of the fluorescent nanofilm. (b) An image of the nanofilm floated on the water. Inset is the fluorescent image of the nanofilm under UV light. (c) Scanning electron microscope (SEM) and (d) transmission electron microscope (TEM) profiles of the nanofilm. (e) Schematic representation of a conceptual sensor and the sensing process for simulated methylamine (MA) leaking, where a 100 mL beaker with 20 mL MA was put into a 50 L box, and the vapor around the liquid was taken out and detected on the sensing platform every 5 min. (f) Fluorescent responses of the nanofilm to MA with different concentrations.

To address these challenges, our team pioneered a dynamic covalent interface self-assembly method to fabricate nanofilms using a tetra-coordinate boron compound, B-24, in combination with benzene-1,3,5-tricarbo-hydrazide (BTH) (Figure 8a).^49^ This method produced nanofilms with highly uniform and porous structures, which enhance mass transfer and minimize substrate effects, leading to improved sensitivity and selectivity in sensing applications (Figure 8b-8d). The nanofilms demonstrated rapid, reversible, and highly sensitive detection capabilities for methylamine (MA), achieving a detection limit below 2.82 mg m^–^^3^ and exhibiting minimal interference from other organic compounds (Figure 8e and 8f). Additionally, the fluorescent films have potential applications in monitoring meat freshness, underscoring their versatility in various practical contexts. However, the sensing mechanism of the nanofilm for methylamine remains unclear, which limits further optimization and broader applications.

**Figure 9:**
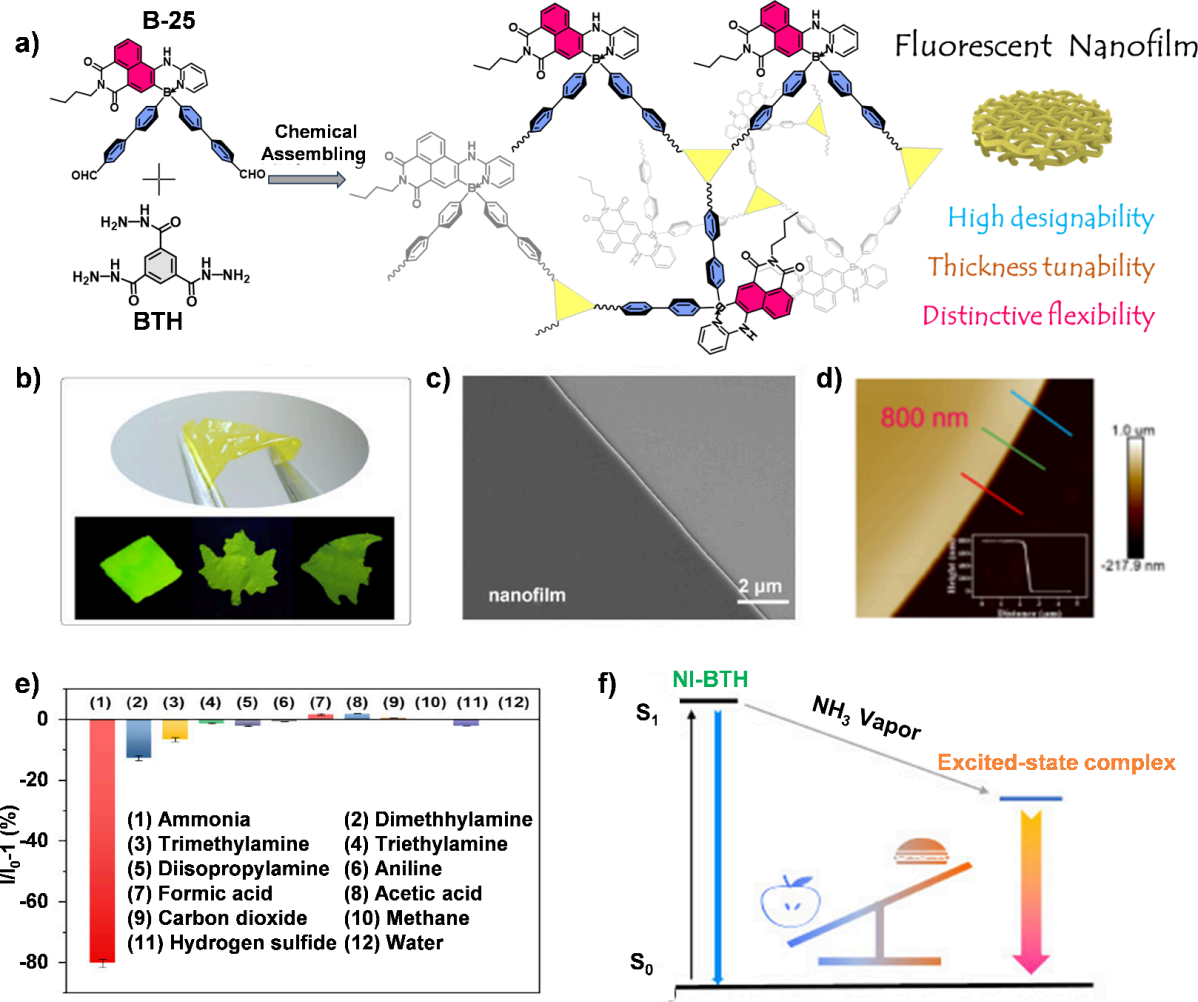
(a) Molecular structures of the two building blocks and schematic illustration of their chemical reaction toward a fluorescent nanofilm. (b) Photographs of the nanofilm folded by tweezers (top) and their fluorescent images with different shapes taken under 365 nm UV light (bottom). (c) SEM image of the B-25@BTH nanofilm on a silicon substrate. (d) AFM images of the nanofilms, where the insets are their height profiles. (e) Fluorescence response of the B-25@BTH nanofilm to NH_3_ vapor and the potential interferences at saturated concentrations. (f) Plausible sensing mechanism of the NI-BTH nanofilm toward NH_3_. Reproduced with permission.^50^ Copyright 2024 American Chemical Society.

Recently, our group developed a nanofilm for the detection of NH_3_ vapor by conducting the dynamic covalent reaction at the air/DMSO interface (Figure 9a) using tetra-coordinate boron compounds (B-25) and benzene-1,3,5-tricarbohydrazide (BTH) as building blocks (Figure 9a).^50^ The nanofilm is smooth, defect-free, tailorable and highly flexible (Figure 9b-9d). The fluorescence of the nanofilm can be significantly quenched upon fumed with NH_3_ (Figure 9e), with a detection limit of less than 0.1 ppm and a rapid response time of 0.2 s. Theoretical studies suggest that the sensing mechanism involves the formation of excited-state complexes through hydrogen bonding interactions between the sensor and the analyte (Figure 9f). This mechanistic insight provides a foundation for the rational design of more efficient sensing materials and may lead to the development of advanced sensors with improved sensing performances.

### 3.2 Anticounterfeiting Applications

With the increasing prosperity of the commodity economy, the issue of counterfeit and inferior products has become increasingly severe, resulting in significant negative impacts on consumers, businesses, and the entire social economy.^51−55^ Therefore, the development of effective anticounterfeiting technologies has become critical. Tetra-coordinate boron compounds, known for their efficient solid-state luminescence, offer a promising avenue for creating multistimulus responsive fluorescent dyes, which can be employed in anticounterfeiting labels. These compounds provide the potential for high-security applications by generating unique luminescent signatures that are difficult to replicate, thereby enhancing the reliability and effectiveness of anticounterfeiting measures.

**Figure 10:**
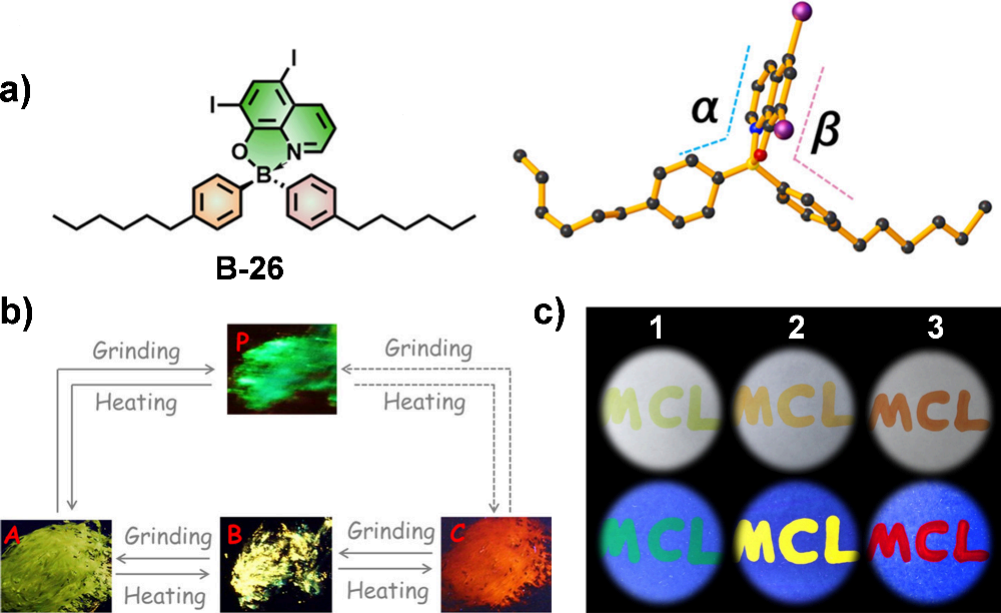
(a) Chemical structure and dihedral angles of B-26. (b) Reversible phase transformation of B-26 in different states as monitored by changes in fluorescent colors (λ_ex_ = 365 nm). (c) Color writing with a single ink (a methyltetrahydrofuran solution of B-26, 1 × 10^–3^ mol L^–1^). (1) Writing with a cool solution at room temperature; (2) writing with a hot solution (>70 °C) at room temperature; and (3) writing with the hot solution on a hot paper. Reproduced with permission.^56^ Copyright 2019 American Chemical Society.

Building on previous research, our team developed a tetra-coordinated boron compound, B-26 (Figure 10a), which is responsive to both mechanical force and thermal stimuli (Figure 10b).^56^ By adjusting the balance between locally excited states and relaxed excited states, we achieved fluorescence color changes from green to yellow to red. This capability led to the innovative development of a single-ink color writing application, demonstrating the compound’s potential for practical use in anticounterfeiting (Figure 10c).

**Figure 11:**
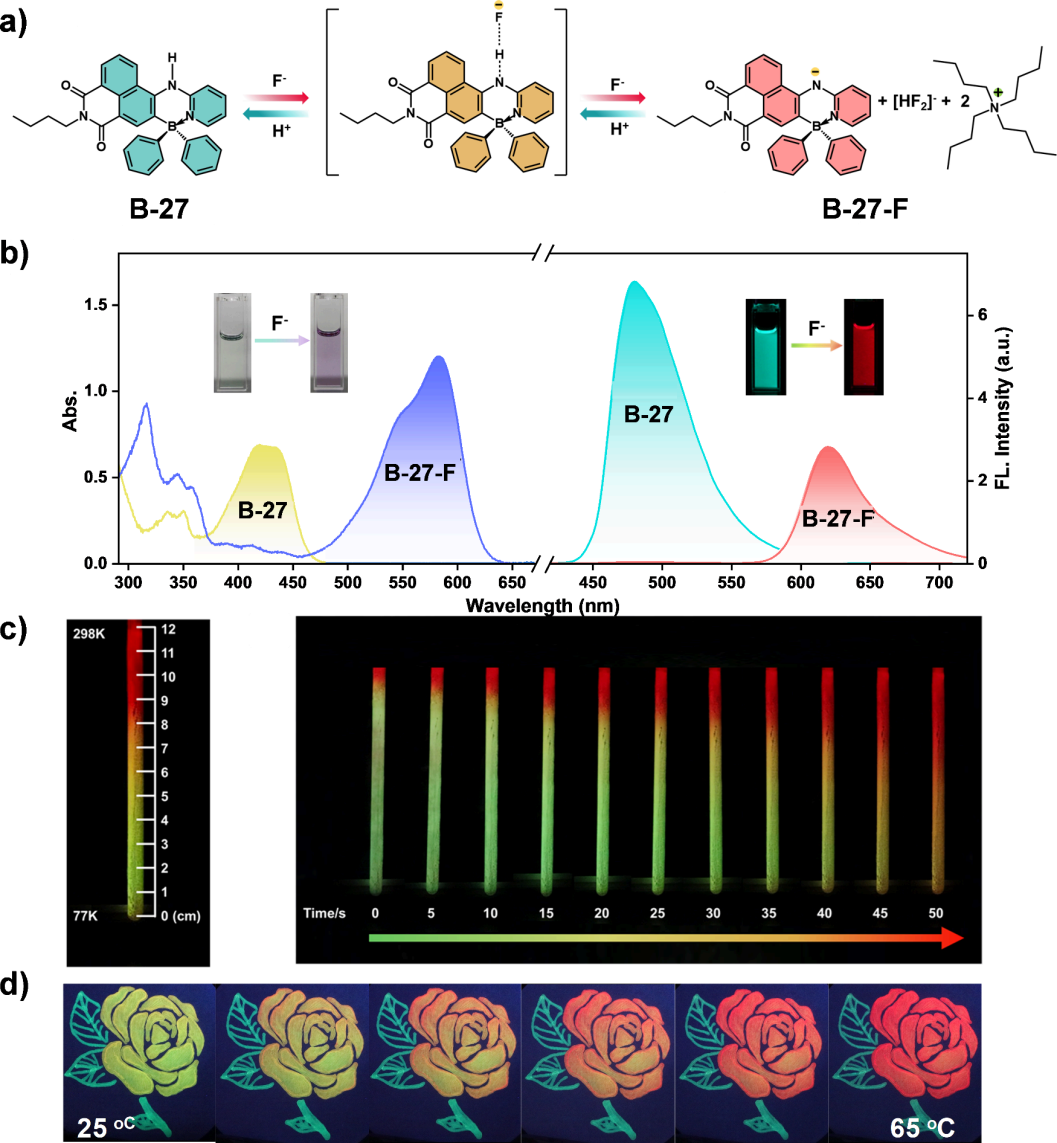
(a) Schematic protonation/deprotonation of B-27. (b) UV–vis absorption and fluorescence emission spectra (λ_ex_ = 365 nm) of B-27 and B-27-F in THF. Inset: the corresponding photographs under daylight and 365 nm UV light (5.0 × 10^–6^ mol L^–1^, 298 K). (c) Photographs of fluorescence color change of a B-27-F solution (1.0 × 10^–4^ mol L^–1^ in THF, 3 equiv. TBAF) in a quartz NMR tube versus temperature gradient and time. (d) Pattern photographs, recorded with B-27 and B-27-F as the encryption ink, before and after heating under ambient light and 365 nm UV lamps, respectively. Reproduced with permission.^57^ Copyright 2022 Wiley-VCH GmbH.

Further inspired by the intelligent luminescence of tetra-coordinate boron compounds, we developed a tetra-coordinate boron compound containing an imine group (B-27). In this system, the imine group (N–H) and tetrabutylammonium fluoride (TBAF) compete for the N–H proton, forming a dynamic fluorescent system (Figure 11a, 11b).^57^ At elevated temperatures, the proton preferentially binds to the fluoride ion, enhancing intramolecular charge transfer within B-27 and changing the color of the system from yellow to purple, accompanied by a fluorescence shift from yellow-green to red. At lower temperatures or in the presence of protic solvents, the N–H bond reforms, reverting the compound to its original state (Figure 11c, 11d). This reversible fluorescence behavior allows for high-level information encryption and decryption in anticounterfeiting labels.

**Figure 12:**
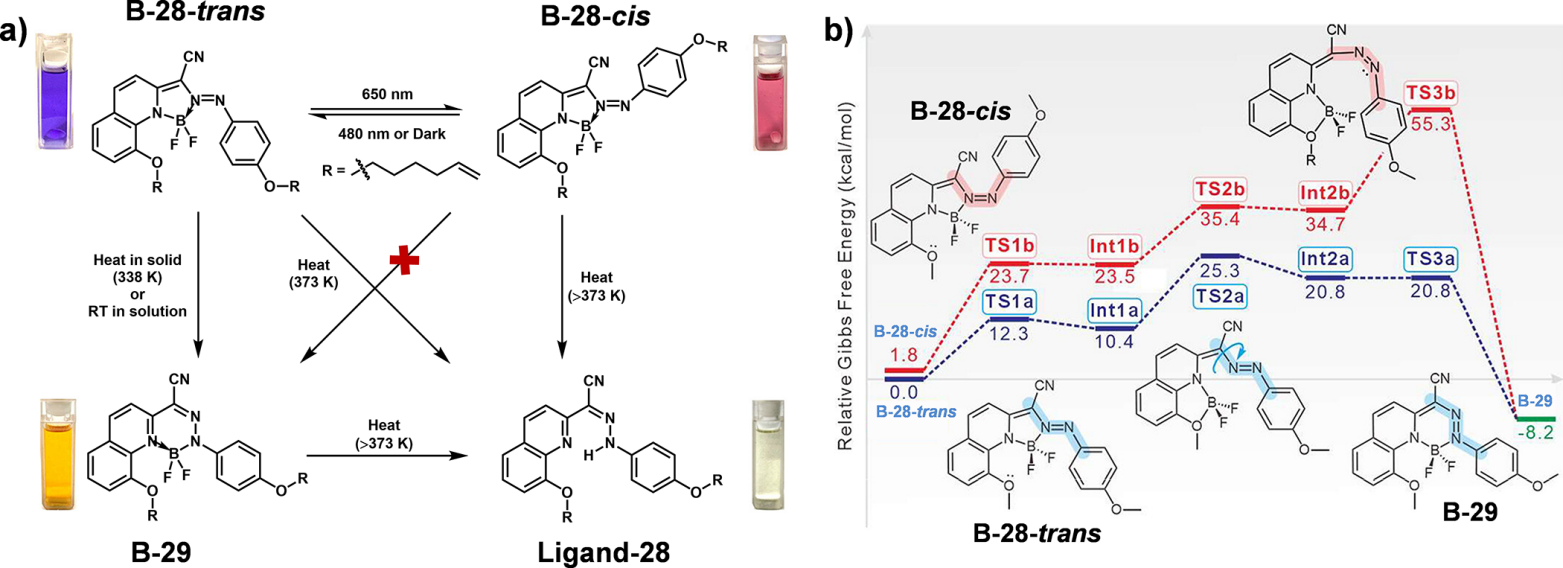
(a) All the possible reaction and isomerization pathways of the 1,2-BF_2_ derivative (B-28) and the corresponding color changes in the solution. The *trans*-form of B-28 (B-28-*trans*) can be photoisomerized to its *cis*-form (B-28-*cis*) under 650 nm irradiation, and thermally isomerized to yield the BODIHY form (B-29). The B-28-*cis* and B-29 can be converted to the hydrazone precursor (Ligand-28). (b) Proposed mechanism for the reaction and isomerization among B-28-*trans*, B-28-*cis* and B-29. The relative activation barriers (Δ*G*^⧧^) and Gibbs free energy differences (Δ*G*°) associated with the isomerization are also shown. Reproduced with permission.^60^ Copyright 2024 American Chemical Society.

Moreover, the vacant *p*_*z*_ orbital of boron can accept lone electron pairs from atoms such as nitrogen, oxygen, or phosphorus, forming metastable B-X (X: N, O or P) bonds that can be easily broken or formed, leading to diverse photophysical properties.^58,59^ Aprahamian et al. demonstrated that the *trans*-form of a 1,2-BF_2_ derivative (B-28-*trans*) can be converted into the BODIHY form (B-29; Figure 12a) in both solid and solution states upon photo or thermal stimulation, resulting in notable color changes. However, the BODIHY form cannot be derived from the *cis*-form (B-28-*cis*; Figure 12a).^60^ Mechanistic studies revealed that the rearrangement was initiated by the lone pairs on the oxygen atom adjacent to the BF_2_ bridge (Figure 12b). Specifically, these lone pairs engaged in a nucleophilic attack on the empty *p*-orbital of the boron atom, leading to the displacement of the nitrogen atom. This interaction would promote the formation of a transient intermediate product (TS2a), where azo nitrogen lone pair can subsequently coordinate with boron. This coordination is critical for the conversion to the BODIHY form, as it helps stabilize the intermediate structure. In this system, such photo- and thermo-switching behavior facilitate multicolor modulation, offering new pathways for developing multiresponsive anticounterfeiting materials with enhanced security features.

### 3.3 Bioimaging and Diagnostics

Organic near-infrared (NIR) fluorescent dyes have gained attention in bioimaging due to their unique optical properties and high biocompatibility, enabling deep tissue imaging for early diagnosis and precision treatment of diseases.^61−65^ Research on tetra-coordinate boron compounds has led to significant advancements in this field, offering several key advantages. First, these compounds can be readily modified to achieve low bandgap, resulting in strong NIR absorption and/or emission. Second, they exhibit high quantum yield and long fluorescence lifetime, enhancing both sensitivity and resolution in imaging applications. Third, their stability and compatibility in complex biological environments make them particularly suitable for *in vivo* applications.

**Figure 13:**
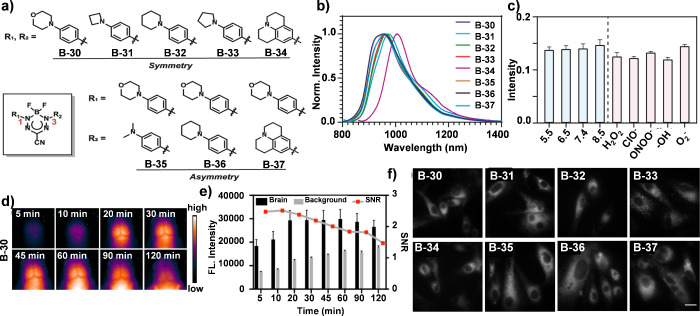
(a) Chemical structures of BF_2_ formazanate dyes B-30 ∼ B-37. (b) The normalized fluorescence spectra of B-30 ∼ B-37 (20 μM) in DMSO. (c) Fluorescent stability of B-30 (10 μM) in PBS (1% FBS) buffer with different pH and ROS species. (d) In vivo NIR-II fluorescence imaging of cerebral tissue of mice injected with B-30 (200 μL, 200 μM, tail vein injection) at time intervals from 5 to 120 min (70 mW cm^–2^, 808 nm laser, 1000 nm LP filter). (e) Time-dependent signal-to-noise ratio (SNR) changes determined by the NIR-II fluorescence imaging of B-30 treated mice. (f) Fluorescence imaging of B-30 ∼ B-37 in U-87 MG cells. All cell imaging experiments were performed using a NIR-II microscope with a 663 nm excitation laser (200 W cm^–2^, exposure 10 ms) and a 950 ± 50 nm emission filter. The scale bar is 10 μm. Reproduced with permission.^66^ Copyright 2022 American Chemical Society.

Recent advancements in optics technologies have expanded the bioimaging window to the NIR-II region (1000–1700 nm), allowing for deeper tissue penetration up to 3–5 cm while maintaining high energy coverage. For example, BF_2_ formazanate fluorophores exhibit a range of rich photophysical properties due to their highly delocalized π-systems and low-lying frontier orbitals, which can stabilize otherwise unstable radicals. Notably, these fluorophores are featured with small molecular sizes, moderate lipophilicity, low polar surface area and minimal charge, suggesting their potential for effective blood-brain barrier (BBB) permeability. Recently, Xiao et al. developed a series of boron difluoride (BF_2_) formazanate NIR-II dyes (Figure 13a) with tunable photophysical properties, exceptional photostability, robust biological stability, and strong fluorescence intensity (Figures 13b, 13c).^66^*In vitro* and *in vivo* imaging studies demonstrated that these dyes can efficiently cross the BBB (Figures 13d-13f), making them suitable for noninvasive NIR-II brain imaging.

**Figure 14:**
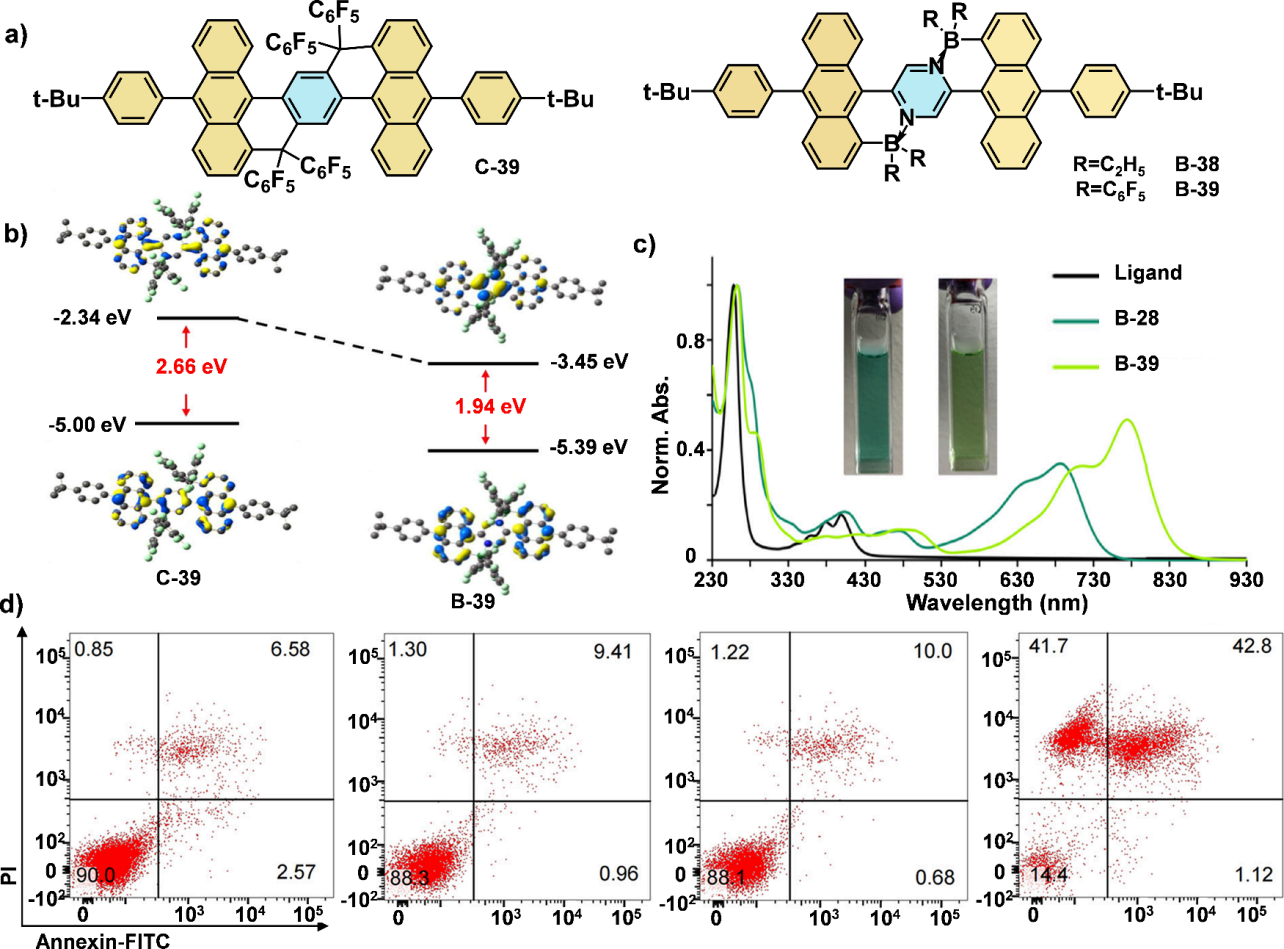
(a) Chemical structure of B–N Lewis pair-fused anthracenes (B-38 and B-39) and the all-carbon analogues (C-39). (b) Computed frontier orbital energies and HOMO/LUMO orbital plots of B-39 in comparison to its all-carbon analogues C-39. (c) UV–vis absorption spectra of Ligand, B-38 and B-39 in DCM. (d) Apoptosis and necrosis analysis using flow cytometry toward 4T1 cells after different treatments. NIR light irradiation (808 nm, 1.0 W cm^–2^, 5 min) was conducted after cells were incubated with B-39-NPs (28 μM). Reproduced with permission.^67^ Copyright 2022 American Chemical Society.

Jäkle et al. reported a novel class of borane-modified dianthracenylpyrazine compounds (Figure 14a),^67^ where the introduction of B–N bonds significantly reduced the HOMO–LUMO gap and notably lowers the LUMO energy compared to their all-carbon analogues (Figure 14b). The B-38 and B-39 derivatives exhibited strong absorption in the NIR region compared to the ligand. Additionally, water-soluble nanoparticles (B-39-NPs) were prepared using the amphiphilic biocompatible copolymer as the encapsulation matrix via the nanoprecipitation method. Figure 14d shows that the percentage of viable cells decreased from 90.0% (PBS) to 14.4%, with over 84% of cells undergoing apoptosis or necrosis after treatment with B-39-NPs under NIR irradiation. In contrast, neither B-39-NPs nor NIR irradiation alone inhibited cell growth. These findings suggest that the B–N Lewis pair functionalization of polycyclic aromatic hydrocarbons (PAHs) holds significant potential for photothermal cancer treatment.

**Figure 15:**
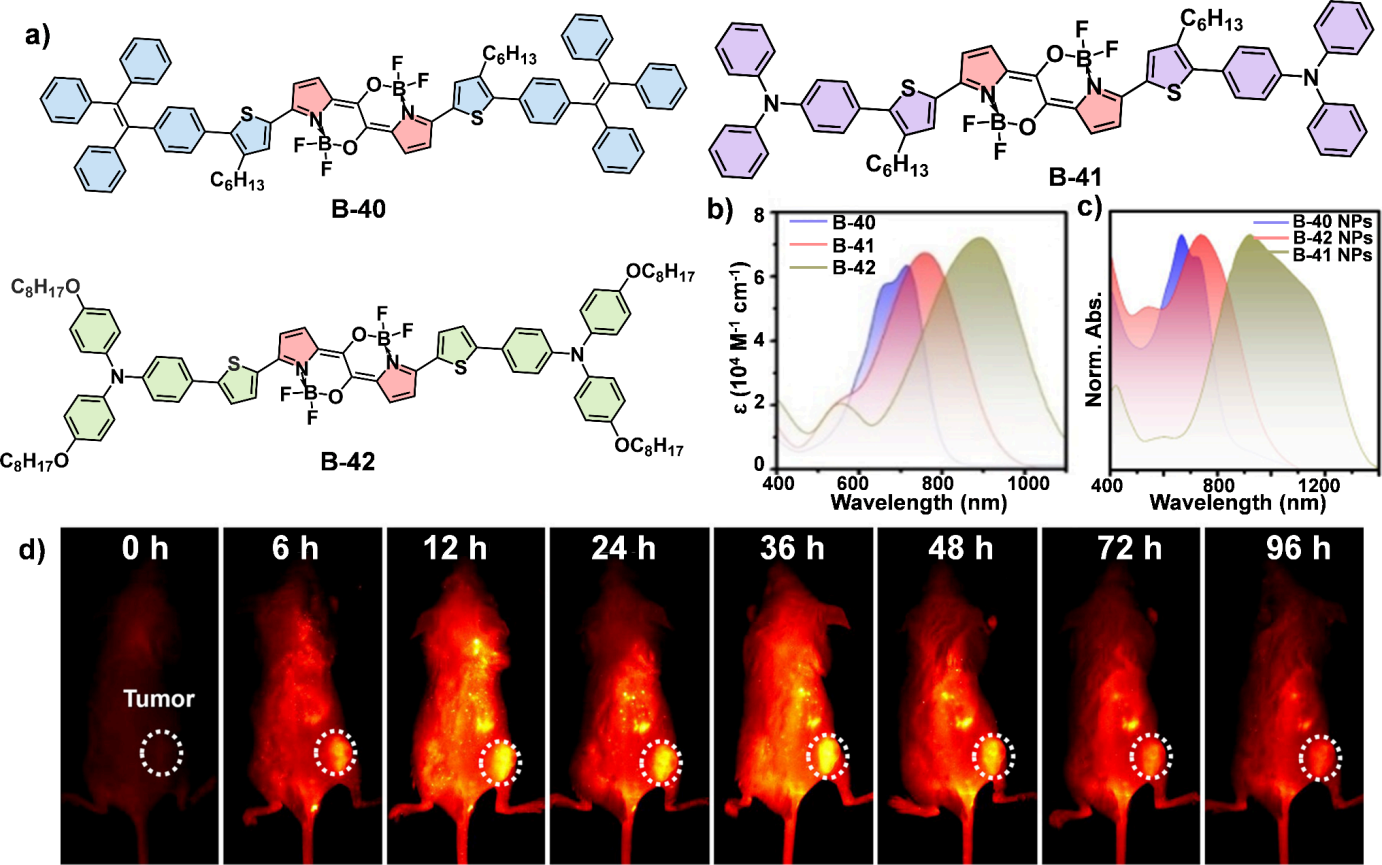
(a) Chemical structure of B-40, B-41 and B-42. (b) Absorption spectra (10 μM) in THF of three compounds. (c) Normalized absorption spectra of B-40 NPs, B-41 NPs, and B-42 NPs in H_2_O. (d) Time-dependent NIR-II fluorescence imaging of tumor-bearing mice after intravenous (i.v.) injection of Cy7-loaded B-42 NPs (1 mg/mL, 100 μL). Reproduced with permission.^68^ Copyright 2022 Wiley-VCH GmbH.

The development of organic photothermal agents capable of absorbing light in the NIR-II region is crucial for the treatment of deep-seated tumors. In a recent study, Tang and colleagues developed three donor–acceptor–donor (D-A-D) structured photothermal agents incorporating strong electron-withdrawing B–N bonds (B-40 ∼ B-42) (Figure 15a).^68^ The absorption wavelength of B-40 and B-41 diminished beyond the 1000 nm region, while that of B-42 extended up to 1100 nm and maintained a high molar extinction coefficient (1.68 × 10^4^ M^–1^ cm^–1^) at 1064 nm (Figure 15b), suggesting the superior light-harvesting ability of B-42 in the NIR-II window. When encapsulated in amphiphilic polymer nanoparticles, B-42 NPs exhibited a broader absorption range (1000–1400 nm) compared to B-40 NPs and B-41 NPs (Figure 15c). Additionally, B-42 retained a high photothermal conversion efficiency (PCE = 54.1%), demonstrating efficacy *in vivo* tumor treatment under 1064 nm laser irradiation (Figure 15d).

## 4 Conclusions and Outlook

This minireview discusses recent advances in tetra-coordinate organoboron compounds as photoactive materials, emphasizing their structural designability and significant impacts on optoelectronic properties. It summarizes four key strategies for tuning the properties of these compounds: ligand modification, adjustment of conjugation length, introduction of multifunctional substituents, and variation of bridging atoms. Through these strategies, we underscore the intricate relationship between molecular design and function. We also detail recent progress in using these compounds for sensing, anticounterfeiting, and bioimaging. The unique electronic properties of these compounds, stemming from the electron-deficient nature of boron, facilitate efficient interactions with conjugated systems, leading to high fluorescence quantum yields, tunable emission wavelengths, and excellent photostability. These properties have been harnessed to develop advanced materials with tailored functionalities for a variety of applications.

In fluorescence sensing, tetra-coordinate boron compounds have demonstrated significant sensitivity and selectivity toward various analytes, making them invaluable for environmental monitoring and health diagnostics. Their unique luminescent properties have also been leveraged for anticounterfeiting applications, offering secure and versatile options for the development of high-level, multiresponsive security features. Additionally, the potential of these compounds in bioimaging, particularly in the NIR-II region, provides promising avenues for high-resolution imaging of deep tissue structures, thus enhancing early disease diagnosis and treatment strategies.

Despite these advancements, several challenges persist in the development and application of tetra-coordinate boron-based photoactive molecules. One major challenge is achieving precise control over their photophysical properties to ensure optimal performance in specific applications. For instance, maintaining high stability and consistent fluorescence properties in varying practical sensing scenarios and biological conditions can be challenging. Moreover, the synthesis of these compounds often involves complex and multistep processes, which can limit scalability and increase production costs. Additionally, the stability of these compounds in biological environments needs further enhancement to ensure their long-term efficacy and safety in clinical applications. Reports indicate that degradation or quenching of fluorescence in physiological conditions can significantly reduce the effectiveness of these materials in bioimaging applications.

Future research should focus on overcoming these limitations by developing novel synthetic strategies that enable more straightforward and cost-effective production while enhancing the stability and functionality of boron-based compounds. Additionally, a deeper understanding of the interaction mechanisms between these compounds and the target analytes or biological environments will be essential to optimize their performance and expand their applicability.

Continued efforts to refine these materials and address their current limitations will be crucial for unlocking their full potential across diverse fields. By enhancing their stability, simplifying their synthesis, and better understanding their interactions in various environments, tetra-coordinate boron-based photoactive compounds could become key players in next-generation optoelectronic devices, medical diagnostics, and secure information systems. Such advancements will not only expand the scope of their applications but also contribute significantly to sensing technological and biomedical innovation.

## Vocabulary

Bidentate chelating ligand: A molecule that uses two donor atoms to form two coordinate bonds with a central atom through two electron-donating atoms (i.e., oxygen, nitrogen, or sulfur), creating a stable ring-like structure called a chelate. These ligands stabilize the coordinated complexes through the chelate effect, which arises from the enhanced stability of cyclic structures and the replacement of multiple monodentate ligands.
Nucleophile: A species that donates an electron pair to form a bond with the electron-deficient center of another chemical species, such as a carbocation or a partially positive center. The strength of a nucleophile depends on factors like charge, electronegativity and the solvent.
Electron delocalization: Electron delocalization refers to the phenomenon where electrons are not confined to a single atom or bond but are spread out over several atoms in a molecule. This occurs in systems with conjugated π bonds or resonance structures, where overlapping *p*-orbitals allow electrons to move freely across the structure. Delocalization increases molecular stability through resonance stabilization and plays a key role in the reactivity and properties of many organic and inorganic compounds.
HOMO–LUMO energy gap: The energy difference between the highest occupied molecular orbital (HOMO) and the lowest unoccupied molecular orbital (LUMO). It determines a molecule’s electronic properties, such as its reactivity, photophysical properties (i.e., absorption, luminescence) and conductivity.
Electron density distribution: Electron density distribution describes how electrons are spatially spread across a molecule or atom. It shows the probability of finding electrons in specific regions, and can be depicted using contour maps or electron clouds. This distribution determines the molecular shape, bond strength and reactivity.
